# Time origin and structural analysis of the induced CRISPR/cas9 megabase-sized deletions and duplications involving the *Cntn6* gene in mice

**DOI:** 10.1038/s41598-019-50649-4

**Published:** 2019-10-02

**Authors:** Inna E. Pristyazhnyuk, Julia Minina, Alexey Korablev, Irina Serova, Veniamin Fishman, Maria Gridina, Timofey S. Rozhdestvensky, Leonid Gubar, Boris V. Skryabin, Oleg L. Serov

**Affiliations:** 1 Institute of Cytology and Genetics, Novosibirsk, 630090 Russia; 2 Novosibirsk State University, Novosibirsk, 630090 Russia; 3 Medical Faculty, Core Facility of Transgenic Animal and Genetic Engineering Models (TRAM), University of Münster, Münster, 48149 Germany; 4 Research Institute of Medical Genetics, Tomsk National Research Medical Center Russian Academy of Sciences, Tomsk, 634050 Russia

**Keywords:** Animal disease models, Genetic engineering, Cytogenetics, Autism spectrum disorders

## Abstract

In a previous study using one-step CRISPR/Cas9 genome editing in mouse zygotes, we created five founders carrying a 1,137 kb deletion and two founders carrying the same deletion, plus a 2,274 kb duplication involving the *Cntn6* gene (encoding contactin-6). Using these mice, the present study had the following aims: (*i*) to establish stage of origin of these rearrangements; (*ii*) to determine the fate of the deleted DNA fragments; and (*iii*) to estimate the scale of unpredicted DNA changes accompanying the rearrangements. The present study demonstrated that all targeted deletions and duplications occurred at the one-cell stage and more often in one pronucleus only. FISH analysis revealed that there were no traces of the deleted DNA fragments either within chromosome 6 or on other chromosomes. These data were consistent with the Southern blot analysis showing that chromosomes with deletion often had close to expected sizes of removed DNA fragments. High-throughput DNA sequencing of two homozygotes for duplication demonstrated that there were no unexpected significant or scale DNA changes either at the gRNA and joint sites or other genome sites. Thus, our data suggested that CRISPR/Cas9 technology could generate megabase-sized deletions and duplications in mouse gametes at a reasonably specific level.

## Introduction

CRISPR/Cas9 (the clustered regularly interspaced repeat associated protein 9) technology is the most attractive approach to perform predictable genome modifications in cells and early embryos of various species^1–5^. As a tool that allows single or few desirable nucleotide changes in targeted genes, this approach has been successfully used for precise correction of point mutations or mutagenesis of investigated genes^1,5–11^. Moreover, directed knock-out (KO) or knock-in (KI) of regulatory elements by CRISPR/Cas9 technology opens a possibility to elucidate their potential functional role in establishment of specific phenotypes. In spite of the small deletions or inversions (as a rule less than 20 bp) to the immediate vicinity of the target site, CRISPR/Cas9 is considered as the most effective and reasonably specific method of genome modification. However, recent data have revealed reasonable grounds for concern about consequences of double-stranded DNA break (DSB) repair^12–14^. DSBs introduced by single-guide RNA/Cas9 endonuclease occur more often than suggested earlier, and they are accompanied by large deletions and other complex genomic rearrangements at the targeted sites in different cell types^12–14^. Similar results have been obtained when large-scale genomic deletions are induced by the CRISPR/Cas9 technique in mouse cells and zygotes^15–22^. Generation of large-scale deletions requires at least two guide-RNAs that produce two DSBs at different sites within the targeted chromosome. In addition, the generation of large-scale deletions is often accompanied by inversions and less often by duplications^18,19,21–24^. Using single strand oligonucleotides (ssODNs) complementary to the most external gRNA sites together with single-guide gRNA targets, the CRISPR/Cas9 approach increases the yield of the predicted targeted deletions^19–21^. Another problem is mosaicisms in distribution of the rearrangements among somatic cells in F0 offspring^19,21,22,25^. In addition, there are limited data concerning origin and future fate of the deleted DNA fragments^15–23^,and estimation of the scale of unpredicted DNA changes accompanying the rearrangements.

Recently, we produced seven mouse founders carrying 1,137 kb deletions involving the *contactin-6* (*Cntn6*) gene by CRISPR/Cas9 technology^21^. Among these founders, we identified two mosaic animals carrying a 2,274 kb duplication involving the same gene. These mice may be useful experimental models for neurodevelopmental disorders since they contain similar chromosomal rearrangements affecting the *CNTN6* gene in human^26–29^. For this reason, it is important to estimate level of unpredicted DNA changes accompanying the rearrangements. The present study is concerned origin and the structural characteristics of these induced CRISPR/Cas9 chromosomal deletions and duplications by FISH analysis, Southern blot hybridization and high-throughput DNA sequencing. No additional significant DNA changes were found to accompany the generation of these targeted megabase-scale deletions and duplication obtained by the CRISPR/Cas9 system.

## Results

### Inheritance of the 1,137 kb deletion and the 2,274 kb duplication among offspring obtained after crossing F0 founders and C57BL6 mice

The inheritance of the 1,137 kb deletion among offspring from three heterozygous founders, namely, #9, #30 and #35, was close to the expected 1:1 ratio (Table 1). These results suggested that the 1,137 kb deletion was generated at the one-cell stage. The increased proportion of mice carrying the 1,137 kb deletion relative to mice with the wild-type genotype among offspring from founder #11 may be explained by insufficient sampling (see below).

**Table 1:** Inheritance of the 1,137 kb deletion and the 2,274 kb duplication among offspring derived from crossing of F0 founders and C57BL mice.

FO founders	Number of crossing	Number of offspring	Genotype of offspring		
			Wild type	Deletion	Duplication
#1**	4	30	19	4	7
#9*	1	8	3	5	
#11*	2	12	3	9	
#15*	2	17	0	17	
#20**	2	20	12	6	2
#30*	2	18	9	9	
#35*	3	18	10	8	

*FO founder-carrier of the 1,137 kb deletion.

**FO founder-carrier of the 1,137 kb deletion together with the 2,274 kb duplication.

All 17 offspring from founder #15 were carriers of this deletion, and there were no animals with a wild-type genotype (Table 1). These data indicated that founder #15 was homozygous for the 1,137 kb deletion, which demonstrated that both homologs of chromosome 6 were targeted. One could expect more loss additional DNA fragments deletions during re-joining of broken DNA ends due to imprecise process of non-homologous end-joining (NHEJ)^1,7,8^. However, sequencing PCR products across the joint site in founder #15 indicated that there was no additional DNA deletions^21^. Moreover, recently we performed additional sequencing of the joint sites in six F1 offspring derived from the founder #15 and the data demonstrated that there was no nucleotide loss at the joint sites of both alleles.

Two founders, namely, #1 and #20, were mosaics carrying both the 1,137 kb deletion and the 2,274 kb duplication, as well as a wild-type allele. Single nucleotide polymorphism (SNP) analysis revealed that both duplications occurred from a C57BL/6 chromosome^21^. The total number of F1 offspring carrying these rearrangements was slightly lower than mice with wild type genotype (Table 1). In addition, the 1,137 kb deletion and the 2,274 kb duplication segregated to progeny from both founders #1 and #20 (Table 1).

### Characterization of the 1,137 kb deletion and 2,274 kb duplication by fluorescence *in situ* hybridization (FISH) analysis of metaphase chromosomes and by Southern blot analysis

**Figure 1:**
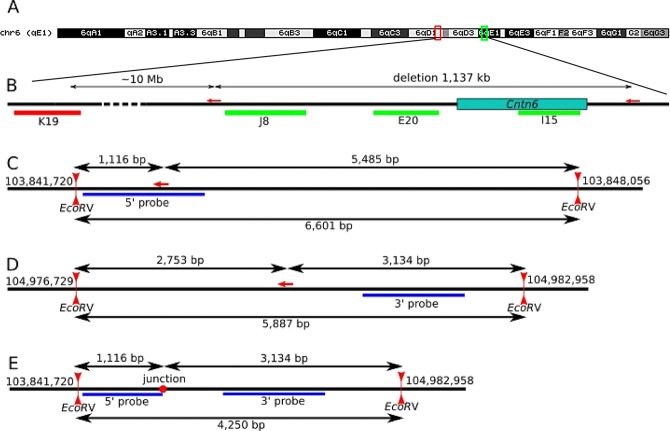
Schematically representation of mouse chromosome 6 (**A**), localizations of BAC clones J8, E20 and I15 (green) along the 1,137 kb deletion including the *Cntn6* gene and K19 clone outside deletion (red) (**B**). Schematically representation of Southern blot analysis for the left and the right sides of allele of wild type (**C**,**D**, respectively) and for a new border of”the deleted allele” (**E**). Vertical red arrowheads according to EcoRV restriction site and genomic coordinates (GRCm38/mm10) mark near each restriction site; horizontal red arrow correspond to CRISPR/Cas9 target site for each gRNA. Blue lines correspond to 5′ probe and 3′ probe for Southern blot analysis. Black double-headed arrows indicate the length of restriction fragments (**C**,**D**). Red point (**E**) indicates the joint site, and genomic coordinates correspond to allele of wild type.

DNA FISH analysis of metaphase chromosomes prepared from primary fibroblasts of five founders carrying the 1,137 kb deletion was performed using the J8, E20, I15 and K19 probes. The J8, E20 and I15 probes (green) were complementary to the total DNA sequence (574732 kb), which covered about half of the 1,137 kb deletion sequence, the K19 probe (red) matched to the genomic coordinates: 93,715727–93,909503 on mouse chromosome 6 that is 10 Mb proximal to the deletion (Fig. 1).

**Figure 2:**
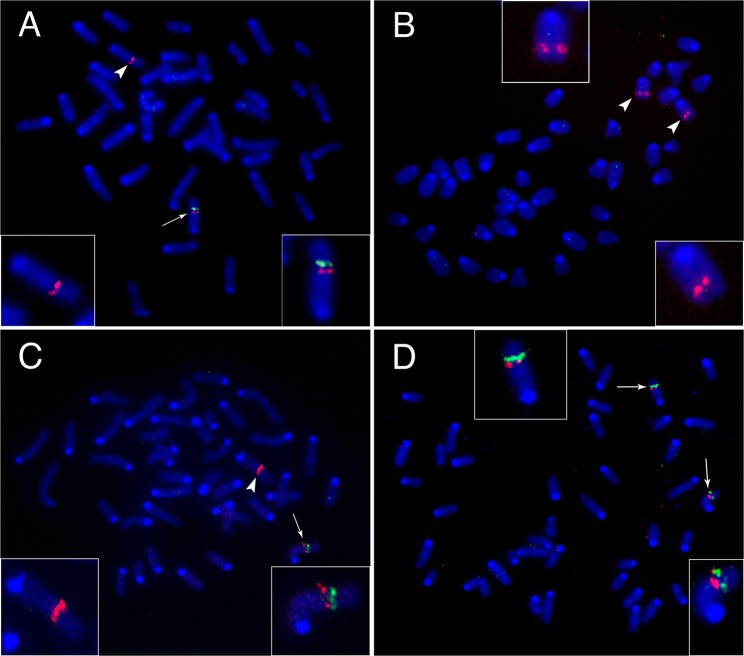
DNA FISH with using of a probe K19 marking a region outside deletion or duplication of mouse chromosome 6 (red) and a probe I15 marking the deleted DNA sequence (green) on metaphase chromosomes of founders: (**A**) #30 heterozygous for the 1,137 kb deletion; (**B**) #15 homozygous for the 1,137 kb deletion; (**C**) #1 and (**D**) #20 carriers both the 1,137 kb deletion and the 2,274 kb duplication. The zoom-in data for targeted chromosome are presented as boxed areas. The arrowhead marks metaphase chromosome 6 with the 1,137 kb deletion whereas arrow indicates chromosome 6 without this deletion.

Both heterozygous and homozygous founder metaphases contained red foci marking both homologs of mouse chromosome 6, whereas green foci were detected either on one homolog (Fig. 2A) or not at all (Fig. 2B). Thus, the inner probes for the deletion reliably identified the presence of the deletion in one or two homologs (Fig. 2; Supplementary Fig. 1). The quantitative distribution of three labeled probes on homologous chromosome 6 derived from mice homo- and heterozygous for the 1,137 kb deletion is presented in Table 2. These data indicated that all four heterozygote founders were not mosaic, which was consistent with the inheritance data (Table 1). The low percentage of positive cells originating from founders #9 and #11 with green foci on both homologs of chromosome 6 resulted from a technical issue.

**Table 2:** Results of FISH analysis with using of probes specific for the 1,137 kb deleted DNA fragment on metaphase chromosomes of fibroblasts derived from the founders carrying chromosomal rearrangements.

Number of FO carrying rearrangement(s)	Number of examined metaphases	Number of metaphases with one labelled homolog of chromosome 6 (%)*	Number of metaphases with both labelled homologs of chromosome 6 (%)**	Absence of FISH signal on chromosome 6 (%)	Cells with trisomy of chromosome 6 (%)***
**Deletion**					
9 ♂	104	101 (97.1)	3 (2.9)	0	
11 ♂	87	85 (97.7)	2 (2.3)	0	
15 ♀	104	0	0	104 (100)	
30 ♀	122	122 (100)	0	0	
35 ♂	100	100 (100)	0	0	
**Deletion and duplication**					
1 ♀	90	31 (34.4)	57 (63.3)	0	2 (2.3)
20 ♀	118	69 (58.5)	46 (39.0)	0	3 (2.5)

*FISH-positive signal (green) on one of the homologs of mouse chromosome 6 only with using of probes I15, J8 and E20 but both homologs contain red label with using by of the K19 probe;

**FISH-positive signals (green and red) on both homologs of mouse chromosome 6 with using of probes I15, J8, E20 and K19;

***Cells with trisomy of chromosome 6 was revealed in fibroblasts derived from the founders #1 and #20 only by K19 probe (red).

Founders #1 and #20 carrying the PCR-detected deletion and duplication of *Cntn6* region were mosaics for the deletion (Table 2; Fig. 2C,D; Supplementary Fig. 1G–I). Founders #1 and #20 had one homolog of chromosome 6 with a deletion in 34.4% and 58.5% examined metaphases, respectively. In the remaining metaphase chromosomes, the inner signal was on both homologs, indicating the presence of normal or duplicated homolog of chromosome 6. Due to the method limitation, it was not possible to distinguish normal and duplicated alleles using DNA FISH on the metaphase chromosomes.

It is important to emphasize that reproducible green foci were not observed in any mouse chromosomes, except chromosome 6, using I15, J8 and E20 probes (Fig. 2; Supplementary Fig. 1). These data demonstrated that none of the DNA fragment(s) derived from the 1,137 kb deleted region were inserted in other mouse chromosomes.

FISH analysis identified 2.3–2.5% of cells with trisomy of chromosome 6 in founders #1 and #20 carrying the duplication (Table 2; Supplement Fig. 1I). Among cells derived from founders #9, #11, #30 and #35 heterozygous and  #15 homozygous for the deletion only, cells with trisomy of chromosome 6 was not find (Table 2). Moreover, the percentage of cells with trisomy will increase to 3.5% and 4.3% in founders #1 and #20, respectively, if do not take into account the cells carrying the deletion together with allele of wild-type. Because it was impossible to distinguish between wild type and duplicated alleles by FISH analysis, the origin of each homolog in these trisomy cells was not determined.

**Figure 3:**
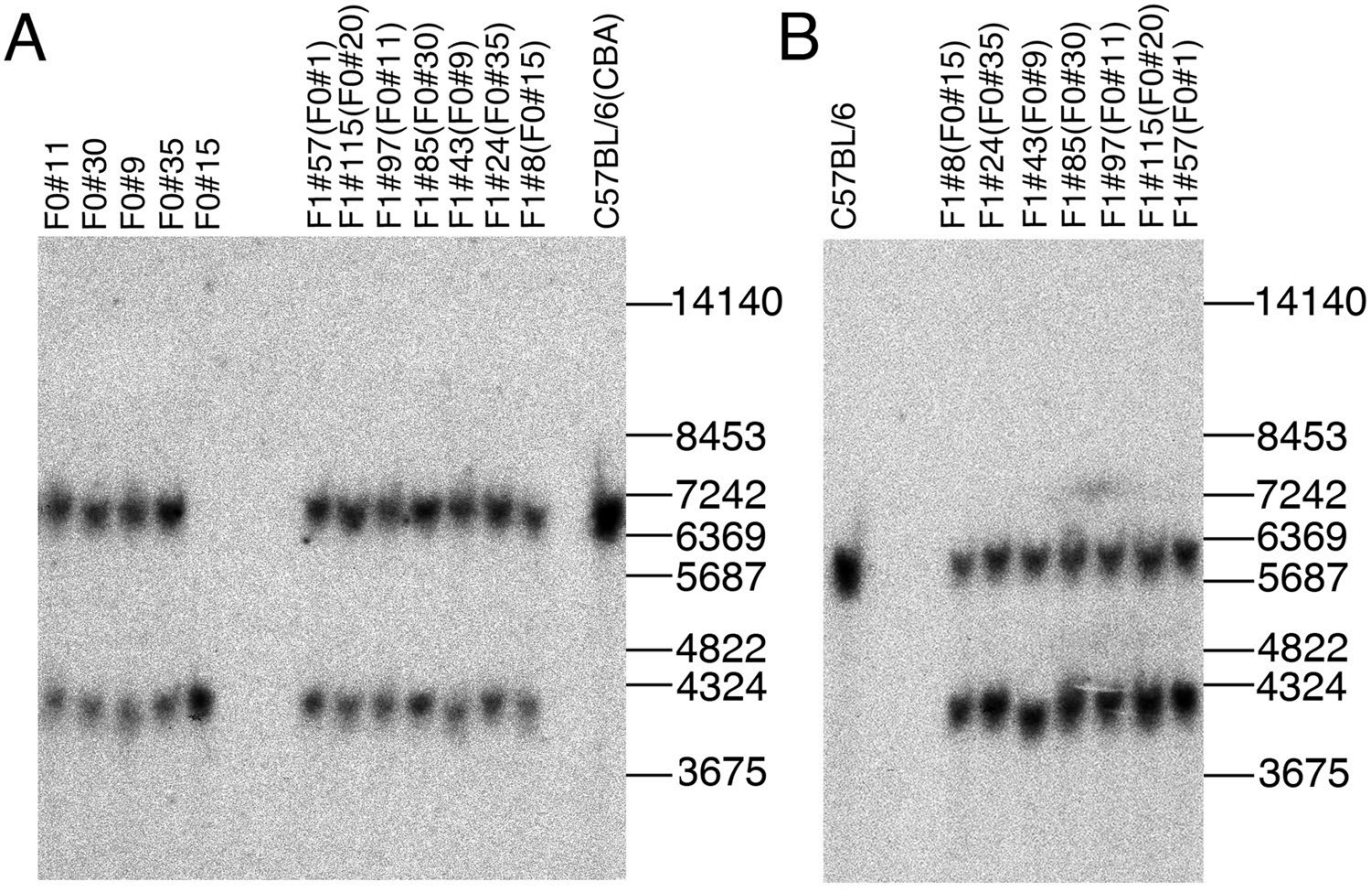
Southern blot analysis of genomic DNA isolated from wt (C57BL/6 and CBA), five FO founders (upper row on the left) carrying the 1,137 kb deletion and seven heterozygous F1 offspring (upper double row on the right) obtained by crossing between all FO founders and C57BL/6 using the 5′-HR probe. (**A**) The two DNA fragments detected by the 5′-HR probe represented the wild type (6,601 bp) and deleted (4,250 bp) alleles. (**B**) Southern blot analysis of C57BL/6 and seven heterozygous F1 offspring (upper double row) derived from crossing between seven FO founders and C57BL/6 mice with using of the 3′-HR probe; the two DNA fragments were identified which represented the wild-type allele (5,887 bp) and deleted allele (4,250 bp). Positions of DNA size marker fragments (in bps) are shown on the right (**A**,**B**).

Figure 3A shows the Southern blot analysis of five F0 founders carrying the 1,137 kb deletion and seven heterozygous F1 offspring from all founders obtained by crossing with C57BL/6 mice. The two DNA fragments detected by the 5′-HR probe represented the wild type (6,601 bp) (Fig. 1C) and deleted (4,250 bp) alleles (Fig. 1E). Importantly, an electrophoretic distance between these DNA fragments are uniform in all analyzed samples, the except FO founder #9 and its F1 #43 progeny in which a DNA fragment derived from the deleted allele has a higher electrophoretic mobility due to a loss of 101 bp at the junction site^21^. Similar results were obtained when analyzing the same heterozygous F1 offspring mice by the Southern blot with using of the 3′-HR probe (Fig. 3B); the two DNA fragments were identified which represented the wild-type allele (5,887 bp) (Fig. 1D) and deleted allele (4,250 bp) (Fig. 1E). Thus, the resolution of the used Southern blot analysis allowed to detect reliably at least 100 bp differences between wild type and the deleted alleles (Fig. 3A,B).

### Characterization of the borders of the 2,274 kb duplication in two animals and high-throughput DNA sequencing

**Figure 4:**
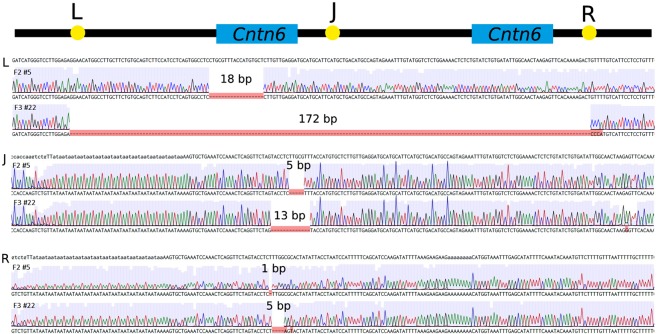
Schematic representation of the 2,274 kb duplication including the *Cntn6* gene and results of Sanger sequencing across the joint sites of left (L), right (R) duplication borders (yellow) and J – junction (J) site (yellow) of duplicated copies. The loss of nucleotides (purple) at the joint sites (as bp) in offspring F2 #5 and F3 #22 derived from founders #1 and #20, respectively.

Our previous study demonstrated that both junction sites between two copies within the 2,274 kb duplication in heterozygous founders #1 and #20 had a deletion of 5 bp and 13 bp, respectively^21^ (Fig. 4).

To identify the left duplication border in a homozygous F2 #5 derived from founder #1, PCR analysis was performed using FWD1.1 and REV1 oligonucleotides and sequencing of PCR products allowed us to identify an 18 bp loss in the duplicated allele (Fig. 4). Using the FWD1.2 and REV1 oligonucleotides, a 172 bp loss was identified in the duplicated allele in a homozygous offspring F3 #22 derived from founder #20 (Fig. 4). In addition, the substitution of a GTTT sequence with CCCA was identified near the 172 bp deletion in the F3 #22 (Fig. 4).

To establish the right border of the duplication, the same homozygous animals, F2 #5 and F3 #22, were studied using the FWD2 and REV2 oligonucleotides in a PCR assay. Sequencing of PCR products demonstrated that the right borders had 1 bp and 5 bp deletions in F2 #5 and F3 #22, respectively. In addition, two mutated nucleotides were identified near the junction site in offspring F3 #22 derived from founder #20 as follows: C at position chr6:104 979 804 was converted to A and C at position chr6:104 979 806 was converted to T (Fig. 4).

High-throughput DNA sequencing results analyzed using the IGV genome browser showed no genomic abnormalities, except the area near borders of the duplicated region noted above. Moreover, the genome sequencing confirmed 18 bp and 1 bp deletions as well as 172 bp and 4 bp deletions at the left and right borders in the homozygous offspring from founders #1 and #20, respectively (Fig. 4). In addition, the genome sequencing confirmed minor changes of nucleotide composition near the left and right borders in an offspring F3 #22 derived from founder #20 presumably due to mutations (Fig. 4).

## Discussion

The present results allow to reconstruct the genome-editing events during generation of the 1,137 bp deletion and the 2,274 bp duplication. Inheritance of the deletion close to 1:1 among offspring of founders #9, #30 and #35 suggested that the rearrangement arose at the one-cell stage. This conclusion was consistent with FISH analysis results detecting up to 100% of examined cells with the deletion located on chromosome 6 (Table 2), which was fully applicable to offspring derived from founder #11. FISH analysis demonstrated that the majority of cells were labeled via FISH on chromosome 6 using specific probes (Table 2; Fig. 2; Supplementary Fig. 1).

Inheritance data of the deleted region among offspring homozygous for the 1,137 bp deletion from founder #15 suggested that the double deletion event arose simultaneously in both the maternal and paternal chromosome 6 at the one-cell stage. Of note, parental homologous chromosomes are localized separately in maternal and paternal pronuclei of one-сell stage fertilized oocytes (stages PN3-PN5 according to Adenot^30^, *i*.*e*., before karyogamy and the first division). The results from the FISH analysis of fibroblasts derived from founder #15 supported this conclusion. All cells were negative for probe sequences of all three labeled BAC clones (Table 2; Fig. 2B).

Simultaneous occurrence of deletion/duplication rearrangements identified in founders #1 and #20 made it possible to determine the mouse embryo developmental stage and preliminary time of CRISPR/Cas9 activity. The ratio of three phenotypes identified among offspring of founders #1 and #20 (Table 1) indicated the segregation of deleted and duplicated alleles. The observed ratio did not differ from the expected Mendelian segregation (Table 1). Hence, these data, together with the SNP analyses, suggest that deletion and duplication occurred at the one-cell stage in a pronucleus carrying a C57BL/6 allele^21^. This suggestion was also supported by FISH analysis of somatic cells from offspring with deletion and duplication. The ratio of cells with deletion, duplication and wild-type allele corresponded to the expected value (Table 2; Fig. 2). The simultaneous occurrence of deletion/duplication events arises due to presumable sister chromatid exchange^19,22,31–33^. The space separation of the parental homologous chromosomes in different pronuclei indicated that deletion occurred after double-strand breaks caused by the CRISPR/Cas9 nuclease in the C57BL/6 allele of chromosome 6, which then generated the 2,274 kb duplication due to an exchange between sister chromatids.

With regard to the fate of the deleted DNA fragment, traces of deleted DNA fragment were not found within chromosome 6 or other mouse chromosomes according to the FISH analysis. Moreover, the FISH analysis of mouse fibroblasts derived from homozygous founder #15 did not reveal any insertions of the deleted DNA fragments.

Similar data have been obtained in a FISH study of megabase-sized deletions (from 0.5 to 5 Mb sizes) generated by CRISPR/Cas9 technology^20–24^. According to fiber-FISH data^34^, however, DNA fragment(s) originating from deletions sometimes can reintegrate close to the excision site. It is important to note that there are technical differences in generation of large-sized deletions. The Boroviak’s protocol^19^ included four gRNAs for targeting additional strand breaks, whereas the present study used only two gRNAs. In addition, fiber-FISH analysis was performed in some founders carrying simultaneous deletions and inversions in the previous study, whereas the present study performed FISH analysis in mice carrying single deletions from independent founders.

Southern blot analysis did not reveal distinctive differences in both 3′- and 5′-flanking sequences, but it detected the 4,250 bp predicted *Eco*RV DNA fragment in all carriers of the 1,137 kb deletion (Fig. 3). The 4,250 bp DNA fragment included sequences around gRNA sites that potentially allowed the identification of structural changes in this region. As mentioned above, loss 101 bp at the joint site in FO#9 was reliably detected due to increasing electrophoretic mobility of the 4,150 bp DNA fragment. Thus, the detected resolution of the used Southern blot analysis (in a range of 4–8 kb) was approximately 2–3%, which permitted substantial genomic rearrangements and aberrations in the perceived region to be revealed. FISH and Southern blot analyses for the 1,137 kb deletion region in five founder mice and their offspring did not detect DNA translocations or significant changes in sequences around gRNA sites.

Another important aspect of CRISPR/Cas9 technology concerns the scale of unpredicted DNA sequence changes during genome editing. Analysis of seven founders carrying the 1,137 kb deletions demonstrated that four of them had minor deletions ranging from a single to dozen bp at rejoining sites, except for founder #9 with a deletion of 101 bp, and three other founders had no deletions at all^21^. Two founders had the 1,137 kb deletion together with the 1,137 kb duplication as well as an additional 13-nucleotide deletion at the rejoining site (Fig. 2). Thus, these data indicated that megabase-sized deletions generated by CRISPR/Cas9 are not accompanied by significant nucleotide rearrangements at the cleavage sites. However, according to Boroviak *et al*.^34^, the excised sequences can reinsert locally. Therefore, short-range junction PCR can lead to an erroneous conclusion.

It is important to consider the high-throughput DNA sequencing data of homozygotes for the 2,274 kb duplication mice. The overall analysis revealed the presence of 24 to 190 bp deletions as well as 2 to 4 bp substitutions surrounding the gRNA-targeting region flanking the duplication. Importantly, these results did not reveal additional genomic abnormalities, except for the region near boundaries of the duplicated area. Thus, despite the complex process of formation of the 2.274 kb duplication, CRISPR/Cas9 genome editing and significant chromosomal aberrations were not observed. However, detection of cells with trisomy of chromosome 6 in two founders harboring the 2,274 kb duplication indicated that a certain degree of chromosomal rearrangements may be responsible for appearance of the trisomy of chromosome 6 in some somatic cells.

In summary, the presented data indicated that CRISPR/Cas9 mediated genome editing could create megabase-sized deletions and duplications in mouse embryos without considerable genomic rearrangements. This conclusion consistent with recent data^35,36^ which showed that CRISPR/Cas9 editing of the mouse genome in zygotes did not accompany a substantial increase in *de novo* mutation frequency. Therefore, CRISPR/Cas9 technology could be a powerful tool for generation of animal models to investigate different human genetic disorders caused by large chromosomal deletions and duplications, including those that disturb the development of the central nervous system that was earlier noted by other investigators^22^.

## Materials and Methods

### Animals and genotyping

The following genotypes of mice were used in the present study: (*i*) seven F0 founders carrying only the 1,137 kb deletion (five animals) or this deletion together with the 2,274 kb duplication (two mosaic mice). Both rearrangements included the entire *Cntn6* gene^21^. Genotyping was performed by PCR as described previously^21^. Primary fibroblasts were obtained from the FO founders carrying the deletion. F2 and F3 mice heterozygous for the 2,274 kb duplication were obtained by repeated backcrossing of founders #1 and #20 with C57BL/6 mice. #F2-5 and #F3-22 mice homozygous for the duplication independent origin were produced by crossing either between F2 heterozygotes originating from #1 or F3 heterozygotes originating from #20, respectively. F2 and F3 mice heterozygous for the 1,137 kb deletion were obtained by repeated backcrossing of F1 and F2 heterozygotes with C57BL/6 mice, respectively.

All mouse procedures complied with the European Communities Council Directive of 24 November 1986 (86/609/EEC) and were approved by the Bioethical Committee at the Institute of Cytology and Genetics (Permission N45 from November 16, 2018). Food and water were available *ad libitum*.

### Production and culture of primary fibroblasts

Preparation of primary fibroblast cultures derived from F0 mice carrying the 1,137 kb deletion was performed as previously described^37^. Briefly, animals were sacrificed by cervical dislocation. The sternum and fingers were isolated, freed of muscles and minced with scissors. Small tissue pieces were placed in a 0.25% trypsin solution and vortexed with glass beads on a magnetic stirrer for 30 min at room temperature. Trypsin was inactivated by adding fetal bovine serum (FBS) in the culture medium. Primary fibroblasts were cultured in DMEM (Gibco; Termo Fisher Scientific Inc) supplemented with 10% FBS (Gibco; Termo Fisher Scientific Inc) and 50 mg/ml penicillin/streptomycin (Gibco; Termo Fisher Scientific Inc) at 37 °C in a 5% CO_2_ atmosphere with high humidity. The medium was changed every 2–3 days.

### Southern blot analysis of the 1,137 kb deletion in FO founders and offspring

Total DNA was isolated from tail tips by digesting in lysis buffer (100 mM Tris-HCl, pH 8.5; 5 mM EDTA; 0.2% sodium dodecyl sulfate (SDS); 200 mM NaCl; and 100 μg/ml Proteinase K (Roche)) overnight at 55 °C. DNA was extracted from the lysates by phenol-chloroform and chloroform treatment, precipitated by isopropanol and washed with 70% ethanol. The DNA pellet was dissolved in TE buffer (10 mM Tris, pH 7.9; and 0.2 mM EDTA). Approximately 5 µg of genomic DNA was digested with *Eco*RV endonuclease (Thermo Scientific™), fractionated on 0.8% agarose gels, and transferred to GeneScreen nylon membranes (NEN DuPont). Membranes were hybridized with a ^32^P-labeled DNA probes specific to 5′- and 3′ - regions flanking the deletion (5′-HR probe and 3′-HR probe, respectively) (Fig. 1). DNA labeling was performed using the random prime DNA labeling kit (Roche), and [^32^P] dCTP (PerkinElmer). Membranes were washed with 0.5x SSPE (1x SSPE is 0.18 M NaCl, 10 mM NaH_2_PO_4_, and 1 mM EDTA, pH 7.7) and 0.5% SDS at 65 °C and exposed to MS-film (Kodak) at −80 °C.

The 3′-HR probe was amplified as two DNA fragments from mouse genomic DNA using the nested PCR approach. The 3′-HR A DNA fragment (0.6 kb) was first amplified using the Cntn6–3hrD1/Cntn6-3hrR1 primer pair and reamplified using the Cntn6-3hrD1pr/Cntn6-3hrR1pr primers (Supplementary Table 1). The 3′-HR B DNA fragment (0.7 kb) was first amplified using the Cntn6-3hrD2/Cntn6-3hrR2 primer pair and consequently reamplified using Cntn6-3hrD2pr/Cntn6-3hrR2pr primers (Supplementary Table 1). The 5′-HR probe was amplified analogously. The 5′-HR A DNA fragment (0.8 kb) was first amplified using the Cntn6-5hrD4/Cntn6-5hrR3 primer pair and reamplified using the Cntn6-5hrD4pr/Cntn6-5hrR3pr primers (Supplementary Table 1). The 5′-HR B DNA fragment (1.0 kb) was amplified using the Cntn6-5hrD3b/Cntn6-5hrR4 primer pair and reamplified using the Cntn6-5hrD3pr/Cntn6-5hrR4pr primers (Supplementary Table 1).

### Preparation and labeling of bacterial artificial chromosome (BAC) probes for FISH analysis

Four BAC clones were purchased from BACPAC Resources Center (California, USA). Three BAC clones, namely, RP24-173I15 (I15; 167390 bp), RP24-85J8 (J8; 218568 bp) and RP23-285E20 (E20; 176350 bp), were complementary to the deleted 1,137 kb DNA fragment of chromosome 6, including the *Cntn6* gene, and RP23-443K19 (K19; 193777 bp) was complementary to the site located approximately 10 Mb proximal to the deleted sequence (Fig. 1A,B). BAC clones were cultured in 100 ml of Luria–Bertani (LB) medium containing chloramphenicol (12.5 μg/ml) at 37 °C for 14–16 h, and they were harvested by centrifugation at 4000 g for 10 min at 4 °C. BAC DNA was extracted and purified from *E*. *coli* via alkaline lysis using phenol-chloroform. The extracted BAC DNA was purified by isopropanol precipitation and dissolved in 100 μl of TE buffer. The DNA concentration was measured using the Qubit dsDNA BR Assay kit (Invitrogen, Life Technologies, USA) on a Qubit 2.0 Fluorometer (Invitrogen, Life Technologies, USA) according to the manufacturer’s instructions. The final DNA concentration was adjusted in the range from 0.1 to 0.2 μg/μl.

The selected BACs were labeled by the Nick translation system (Invitrogen) using digoxigenin-11-dUTP (Rochem Manncheim, Germany) and Biotin-dUTP (BioSet; Novosibirsk, Russia) according to the Roche protocol.

### FISH analysis of mouse chromosome 6 containing either the 1,137 kb deletion or the 2,374 kb duplication

The metaphase spreads were prepared as described earlier^37^. Briefly, cells were treated with 20 ng/ml colcemid during a 2 h prior to hypotonic shock with 0.075 mM KCl for 20 min at 37 °C and then fixed in methanol:acetic acid (3:1).

FISH was performed according to a previously described protocol^38^ with minor modifications. Briefly, after a 1 h treatment with RNAse A (100 µg/ml) and three washes with 2x SSC buffer, slides were subjected to pepsin (0.02% in 10 mM HCl) for 5 min at 37 °C. Slides were then fixed in 1% paraformaldehyde in phosphate buffer saline (PBS) for 10 min and denatured in 70% formaldehyde for 2 min at 75 °C. DNA probes were denatured for 5 min separately at 95 °C and then subjected to prehybridization for 50 min at 37 °C in the presence of Cot1 DNA. After hybridization at 37 °C in a humid chamber for 3 days, biotin BAC probes were detected with the ExtrAvidin-FITC conjugate (1:100; E2761; Merck, Darmstadt, Germany) and goat anti-avidin–FITC antibody (1:100; SP-2040; Vector Laboratories, Burlingame, CA, USA), and digoxigenin-labeled BAC probes were detected with sheep anti-digoxigenin antibodies conjugated with rhodamine (1:100; Roche; Manncheim, Germany) and rabbit polyclonal to sheep antibodies conjugated to rhodamine TexasRed (1:100;Abcam, Cambridge, UK). Chromosomes were counterstained with 4,6-diamidino-2-phenylindole (DAPI) (Merck, Darmstadt, Germany). Microscopic analysis and image capturing were performed using an Axioplan 2 imaging microscope (Zeiss, Germany) equipped with a CV-M300 CCD camera (JAI Corporation, Japan) and ISIS5 software (METASystems). GmbH). Microscopy was performed at the Multiple-Access Center for Microscopy of the Institute of Cytology and Genetics of SB RAS (Novosibirsk, Russia). Approximately 100 metaphase spreads were analyzed for each fibroblast culture.

### High-throughput DNA sequencing of two carriers of the 2,274 kb duplication of independent origin and characteristics of duplication borders

Establishment of the left and right borders of the duplication in two homozygous offspring F2 #5 and F3 #22 derived from founders #1 and #20, respectively, was done by PCR followed by sequencing of PCR products. To study the left (L) border (Fig. 4), the FWD1.1 (TGGTAGGGTGTAACCTTGGGA) and REV1 (TGCACATGACCCATGACCTC) primers were used for PCR of an offspring F2 #5 from founder #1, and the FWD1.2 (TGGGTCCTTGGAGAGGAACA) and REV1 primers were used for PCR of an offspring F3 #22 from founder #20. To study the right (R) border (Fig. 4), the FWD2 (TCCCCATCTGCTGGCTCTAT) and REV2 (CCCCCAAGTGATGCTTCTGT) primers were used for PCR of both homozygous offspring from founders #1 and #20, respectively. PCR products were separated by electrophoresis in 2% agarose gels. DNA fractions of suitable sizes were excised from the gel, and both ends were sequenced as described previously^21^.

The F2 #5 and F3 #22 homozygous animals carrying the duplication originating from the second and the third generations derived from founders #1 and #20, respectively, were subjected to high-throughput DNA sequencing.

Genomic DNA was extracted from tail tips as described above. An Illumina library was prepared, and sequencing was performed using the commercial GeneWiz service (New Jersey, USA). Sequencing was performed using 150 × 2 paired-end chemistry yielding approximately 400 and 460 million reads for #F2-5 and #F3-22, respectively. Raw reads were analyzed using *fastqc* software to ensure high data quality and aligned to mouse *mm10* reference (based on the genome of C57BL/6 mice) using bowtie2 with default parameters. To perform data visualization, alignments were converted to bam format, sorted, indexed using *samtools* and loaded as the IGV browser track. The sequencing data is available in the NCBI under accession PRJNA525906.

## Supplementary information

Dataset 1
