# Supplementary material for: Time origin and structural analysis of the induced CRISPR/cas9 megabase-sized deletions and duplications involving the Cntn6 gene in mice
Source: Sci Rep. 2019 Oct 2;9:14161. doi: 10.1038/s41598-019-50649-4 (PMC6775113; doi:10.1038/s41598-019-50649-4)
Supplement: Supplementary file 1 — Dataset 1 [file 41598_2019_50649_MOESM1_ESM.pdf]

Time origin and structural analysis of the induced CRISPR/cas9 megabase-sized deletions and duplications involving the *Cntn6* gene in mice

Inna E. Pristyazhnyuk[1,4*], Julia Minina[1,4*], Alexey Korablev[1,4], Irina Serova[1], Veniamin Fishman[1,2], Maria Gridina[1,4], Timofey S. Rozhdestvensky[3], Leonid Gubar[3], Boris V. Skryabin[3], Oleg L. Serov[1,2,4**]

[1] – Institute of Cytology and Genetics, Novosibirsk, 630090, Russia

[2] - Novosibirsk State University, Novosibirsk, 630090, Russia

[3] - Medical Faculty, Core Facility of Transgenic Animal and Genetic Engineering Models (TRAM), University of Münster, Münster, 48149, Germany

[4] - Research Institute of Medical Genetics, Tomsk National Research Medical Center Russian Academy of Sciences, Tomsk, 634050, Russia

Supplementary Fig.1

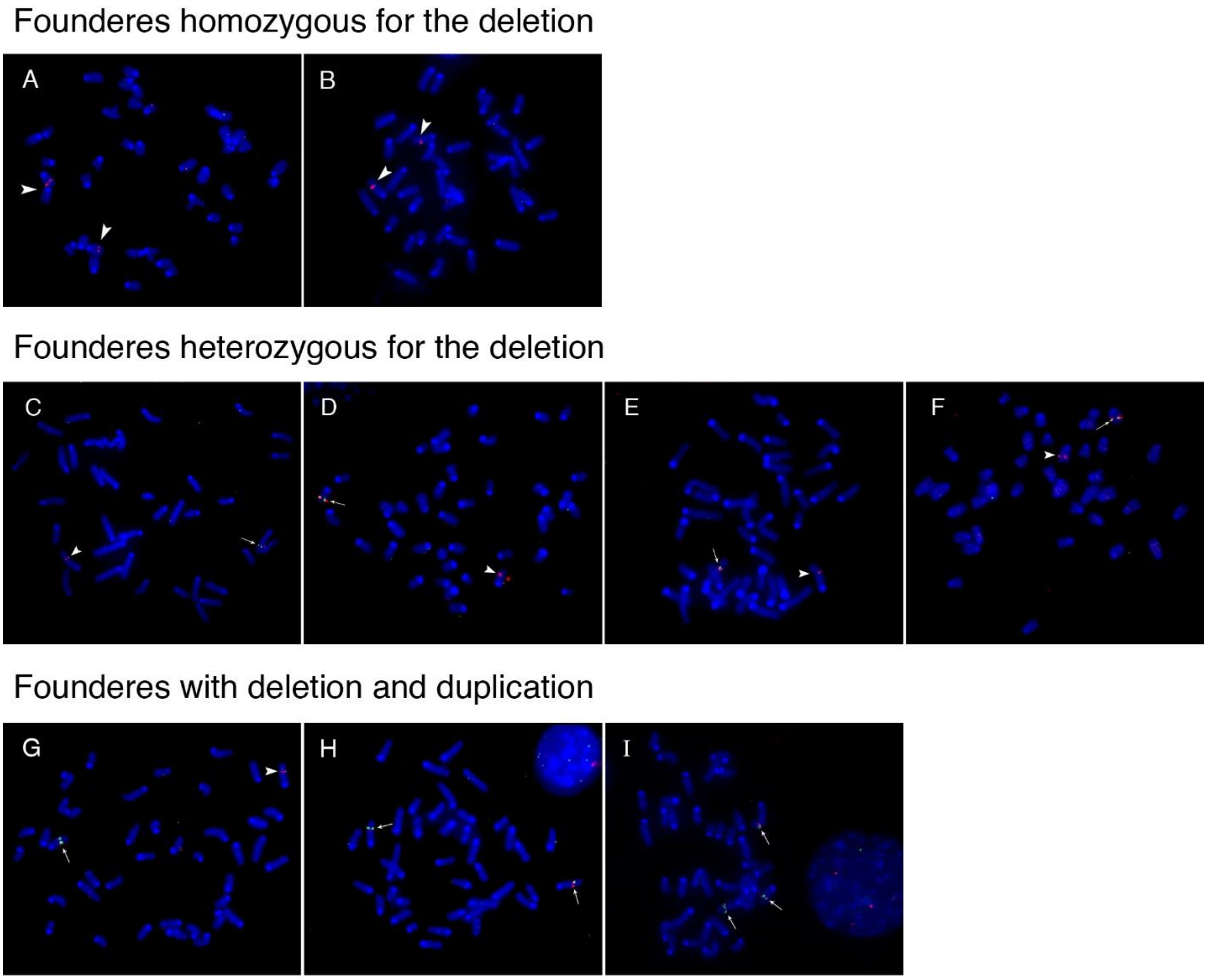

Legends to Supplementary Figures

Supplementary Fig.1

DNA FISH analysis of metaphase spreads of FO founders using a probe K19 (red) specific for mouse chromosome 6 and probes I15, J8 and E20 (green) marking the 1,137 kb deletion only. Upper row – #15 homozygous for the deletion analyzed by probes J8 (A) and E20 (B). Middle row – #9 (C), #11 (D), #30 (E) and #35 (F) heterozygous for the deletion analyzed by probes J8, I15, E20 and J8, respectively. Bottom row - #20 (G) and #1 (H) carrying simultaneous deletion and duplication were analyzed using probes E20 and J8, respectively. A metaphase plate of founder #20 (I) with trisomy for chromosome 6. The arrowhead marks the homolog with the deletion and arrow indicates the homolog without the deletion.
